# Immunomodulation in Respiratory Syncytial Virus Infection: Mechanisms, Therapeutic Targets, and Clinical Implications

**DOI:** 10.3390/microorganisms13081876

**Published:** 2025-08-12

**Authors:** Vasiliki Epameinondas Georgakopoulou, Vassiliki C. Pitiriga

**Affiliations:** 1 Department of Pathophysiology, Laiko General Hospital, National and Kapodistrian University of Athens, 17 Agiou Thoma Street, 11527 Athens, Greece; 2 Department of Microbiology, Medical School of Athens, National and Kapodistrian University of Athens, 75 Mikras Asias Street, 11527 Athens, Greece

**Keywords:** respiratory syncytial virus (RSV), innate immunity, adaptive immune response, immune evasion, immunopathology

## Abstract

Respiratory syncytial virus (RSV) remains a leading cause of acute lower respiratory tract infections globally, particularly affecting infants, older adults, and immunocompromised individuals. While recent advances in prophylaxis, such as long-acting monoclonal antibodies and maternal immunization, offer promise for prevention, therapeutic options for active infection remain limited. Severe RSV disease is often driven not solely by viral replication but by dysregulated host immune responses, including excessive cytokine production, T helper type 2 (Th2) and T helper type 17 (Th17) cell polarization, and impaired interferon signaling. RSV has evolved sophisticated immune evasion strategies, such as inhibition of dendritic cell maturation, degradation of signal transducer and activator of transcription 2 (STAT2) via nonstructural proteins 1 and 2 (NS1/NS2), and interference with pattern recognition receptor signaling, particularly Toll-like receptors (TLRs) and retinoic acid-inducible gene I (RIG-I)-like receptors. These mechanisms result in attenuated innate immune responses and defective adaptive immunity, contributing to viral persistence, immunopathology, and recurrent infections. Moreover, age-dependent vulnerabilities, such as immune immaturity in infants and immunosenescence in older adults, exacerbate disease severity. Excessive immune activation leads to bronchiolitis, airway remodeling, and long-term sequelae including wheezing and asthma. Emerging immunomodulatory therapies aim to restore immune balance, targeting cytokines (e.g., interleukin-6 [IL-6], interleukin-1 beta [IL-1β]), the Janus kinase–signal transducer and activator of the transcription (JAK-STAT) pathway, or inflammasome activity. Host-directed therapies and direct-acting antivirals are also under investigation. A better understanding of RSV–host immune interactions is critical for optimizing therapeutic strategies and designing effective vaccines. This review synthesizes current knowledge on RSV immunopathogenesis and highlights immunomodulation as a promising frontier for therapeutic intervention.

## 1. Introduction

Respiratory syncytial virus (RSV) is a leading cause of acute lower respiratory tract infections (LRTIs), especially among infants, the elderly, and immunocompromised individuals. Globally, RSV is estimated to cause approximately 33 million LRTIs annually in children under five years of age, leading to more than 3 million hospital admissions and around 100,000 deaths [1]. In older adults (≥65 years) and patients with underlying cardiopulmonary or immunosuppressive conditions, RSV contributes significantly to morbidity and mortality, comparable to that seen with non-pandemic influenza [2].

RSV infection displays a well-characterized seasonal distribution, with peak incidence during the colder months in temperate regions and during the rainy season in tropical climates. Transmission occurs primarily via respiratory droplets and fomites, and the high contagiousness of the virus leads to frequent outbreaks [3]. Despite repeated exposures throughout life, RSV does not induce durable sterilizing immunity, making reinfections common, even in adulthood [3]. This is due to multiple factors, including rapid waning of mucosal IgA [4], limited T cell memory [5], and active viral immune evasion via nonstructural proteins nonstructural protein 1 (NS1) and nonstructural protein 2 (NS2) and SH proteins [6,7]. Additionally, RSV suppresses germinal center activity and affinity maturation [8], while infants show Th2-skewed responses, and elderly individuals experience immunosenescence [9,10].

Clinically, RSV infection presents along a spectrum. In infants and young children, it is the most common cause of bronchiolitis and viral pneumonia, with symptoms including cough, wheezing, hypoxemia, and respiratory distress [11]. Severe cases may require hospitalization, mechanical ventilation, or intensive care support. In older adults and individuals with chronic conditions, RSV can exacerbate underlying diseases such as chronic obstructive pulmonary disease (COPD) or congestive heart failure, leading to significant healthcare utilization [12].

Recent advances have led to the approval of the first active immunization strategies against RSV, including vaccines targeting older adults and pregnant women to protect infants via transplacental antibody transfer [13,14,15]. In addition, monoclonal antibodies with extended half-lives are now available for the passive immunization of neonates during RSV season [16]. These developments represent major milestones in RSV prevention after decades of research setbacks.

However, therapeutic options remain limited, as no broadly approved antiviral treatments exist, and current clinical management is mainly supportive. Moreover, severe disease is often driven not solely by viral replication, but by the host’s dysregulated immune response, characterized by excessive cytokine production, airway inflammation, and impaired resolution [17]. RSV has evolved multiple strategies to modulate and evade innate and adaptive immunity, including suppression of type I interferon responses, alteration of dendritic cell function, and skewing of T helper cell responses toward a T helper type 2 cell (Th2)/T helper type 17 cell (Th17) profile [18].

Given this complexity, there is growing interest in immunomodulation as a therapeutic approach, either to enhance antiviral defenses in the early phase or to mitigate immune-mediated lung injury in severe cases. Investigating the mechanisms of immune activation and suppression during RSV infection is essential to guide the rational development of host-directed therapies, vaccine adjuvants, and disease-modifying interventions.

## 2. Immune Response to RSV Infection

**Figure 1:**
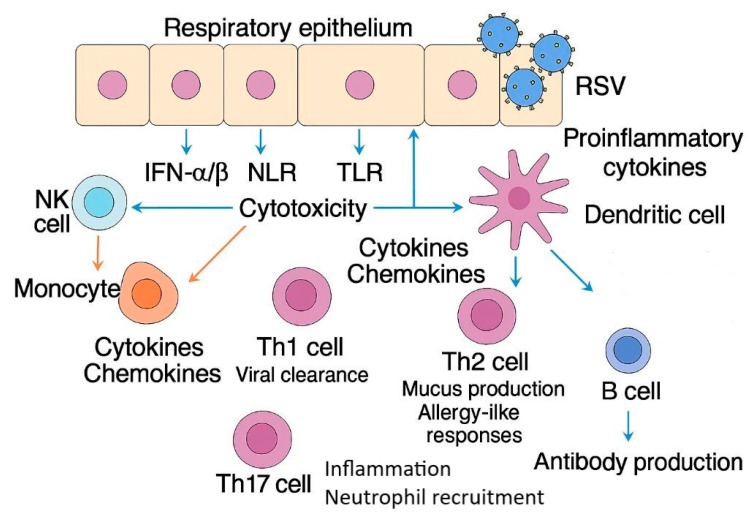
Schematic overview of the innate and adaptive immune responses to RSV infection. RSV is recognized by respiratory epithelial cells through pattern recognition receptors (PRRs), including RIG-I-like receptors (RLRs), NOD-like receptors (NLRs), and Toll-like receptors (TLRs). This triggers the production of type I interferons (IFN-α/β) and proinflammatory cytokines, leading to the recruitment and activation of natural killer (NK) cells, monocytes, and dendritic cells. Dendritic cells, in turn, activate the following CD4+ T helper cell subsets: Th1 cells (promoting viral clearance), Th2 cells (involved in mucus production and allergy-like responses), and Th17 cells (inflammation and neutrophil recruitment). The figure does not depict the role of CD8+ cytotoxic T lymphocytes (CTLs), B cells, T regulatory cells (Tregs), or key viral evasion mechanisms described in the text and, thus, does not fully represent the complexity of RSV immunopathology.

The immune response to RSV infection involves a complex interplay between innate and adaptive mechanisms (Figure 1).

Upon RSV infection of the respiratory epithelium, the host immune system initiates a cascade of responses mediated by pattern recognition receptors (PRRs), including RIG-I-like receptors (RLRs), NOD-like receptors (NLRs), and Toll-like receptors (TLRs). RLRs (such as RIG-I and MDA5) detect viral RNA in the cytoplasm and trigger MAVS-dependent signaling that results in the production of type I interferons (IFN-α/β), which enhance antiviral defenses and activate natural killer (NK) cells. NLRs, including NOD2 and NLRP3, detect cytoplasmic danger signals and contribute to inflammasome assembly, promoting IL-1β release and amplifying inflammation. TLRs such as TLR3 and TLR7 recognize double-stranded or single-stranded RSV RNA in endosomes and further stimulate pro-inflammatory cytokine production. These signals lead to the recruitment of innate immune cells, including NK cells and monocytes, as well as the activation and migration of dendritic cells (DCs) to regional lymph nodes. There, DCs present RSV antigens to naïve CD4+ T cells, directing their differentiation into Th1, Th2, or Th17 subsets. Th1 responses support viral clearance, whereas Th2 and Th17 polarization is associated with exaggerated inflammation, mucus hypersecretion, and in severe cases, lung injury. In parallel, B cells activated by DCs and CD4+ T cells produce RSV-specific antibodies, contributing to neutralization and long-term humoral defense, albeit often short-lived.

### 2.1. Innate Immune Response

The innate immune system constitutes the first line of defense against RSV, initiating antiviral responses and shaping downstream adaptive immunity. Upon infection of airway epithelial cells, RSV is sensed by various PRRs that detect viral components and replication intermediates.

#### 2.1.1. Recognition by Pattern Recognition Receptors (PRRs)

RSV is detected by membrane-bound and cytoplasmic PRRs, including Toll-like receptors (TLRs), RIG-I-like receptors (RLRs), and NOD-like receptors (NLRs). Among TLRs, TLR3 recognizes double-stranded RNA (dsRNA), TLR4 binds the RSV fusion (F) protein, and TLR7 detects single-stranded RNA (ssRNA) within endosomes [19,20]. In parallel, the cytosolic RLRs, retinoic acid-inducible gene I (RIG-I) and melanoma differentiation-associated protein 5 (MDA5), sense viral RNA species and activate mitochondrial antiviral-signaling protein (MAVS), which leads to the downstream activation of transcription factors NF-κB and interferon regulatory factors 3 and 7 (IRF3/7) [21].

These signaling pathways culminate in the production of type I (IFN-α/β) and type III interferons (IFN-λ), which establish an antiviral state via the induction of interferon-stimulated genes (ISGs) such as MxA, OAS1, and ISG15. These ISGs limit viral replication, degrade viral RNA, and enhance antigen presentation [20,22].

#### 2.1.2. Innate Immune Effector Cells

Activated PRRs drive the recruitment of multiple innate immune cell subsets to the infected airway. Dendritic cells (DCs), especially plasmacytoid DCs, are a primary source of type I IFNs. Conventional DCs phagocytose viral particles and migrate to draining lymph nodes, where they prime naïve T cells [23]. RSV impairs DC maturation, reducing co-stimulatory molecule expression and MHC class II presentation, thereby weakening T cell activation [8].

Alveolar macrophages contribute to viral clearance via phagocytosis and cytokine secretion, including TNF-α, IL-6, and IL-1β, and generate reactive oxygen species, while protective, excessive macrophage activation contributes to immunopathology [24].

Neutrophils are rapidly recruited and dominate the inflammatory milieu in RSV-infected lungs. They release proteolytic enzymes such as elastase and myeloperoxidase, as well as neutrophil extracellular traps (NETs), which aid in viral control but also inflict epithelial damage and worsen disease severity if unregulated [25].

Natural killer (NK) cells are recruited to the lungs early during RSV infection, executing cytotoxic functions through perforin and granzyme release and secreting IFN-γ to support the antiviral response. RSV-infected epithelial cells upregulate NK cell-activating ligands, but viral proteins such as NS1 and NS2 interfere with NK activity by modulating MHC-I expression and cytotoxicity [26,27].

Innate lymphoid cells (ILCs), especially group 2 ILCs (ILC2s), respond to epithelial-derived alarmins, including IL-33, IL-25, and thymic stromal lymphopoietin (TSLP). Activated ILC2s secrete IL-5 and IL-13, promoting eosinophilic inflammation, mucus production, and airway hyperresponsiveness. Although initially protective, these responses contribute to immunopathology and are associated with asthma development [28,29].

#### 2.1.3. Inflammasome Activation and NOD2-Mediated Inflammatory Signaling

Beyond classical PRR signaling, the NLRP3 inflammasome plays a central role in sensing cellular stress and viral components. RSV activates NLRP3 in epithelial cells and monocytes, leading to assembly of the inflammasome complex, caspase-1 activation, and secretion of IL-1β and IL-18 [30,31]. These cytokines promote neutrophil infiltration, increase vascular permeability, and amplify inflammation. However, excessive or prolonged inflammasome activation contributes to airway injury and bronchial hyperreactivity [30].

In addition to NLRP3, the nucleotide-binding oligomerization domain-containing protein 2 (NOD2) also plays a crucial role in the innate immune response to RSV. NOD2 is a cytosolic PRR that typically recognizes muramyl dipeptide (MDP), a component of bacterial peptidoglycan, but it has also been shown to detect viral single-stranded RNA, including that of RSV. Upon activation, NOD2 signals through receptor-interacting serine/threonine-protein kinase 2 (RIPK2), leading to NF-κB activation and the induction of pro-inflammatory cytokines such as IL-6 and TNF-α. Notably, NOD2 activation in airway epithelial cells and monocytes during RSV infection enhances the production of chemokines like CCL2 and CXCL10, promoting leukocyte recruitment and amplifying local inflammation. Experimental data suggest that NOD2 contributes to both antiviral defense and immunopathology, as its overactivation can exacerbate pulmonary inflammation and airway hyperresponsiveness. Therefore, NOD2 represents a parallel and complementary pathway to NLRP3 in shaping the RSV-induced innate immune response and should be recognized as a critical component of inflammasome-independent inflammation in this context [32,33,34,35].

**Figure 2:**
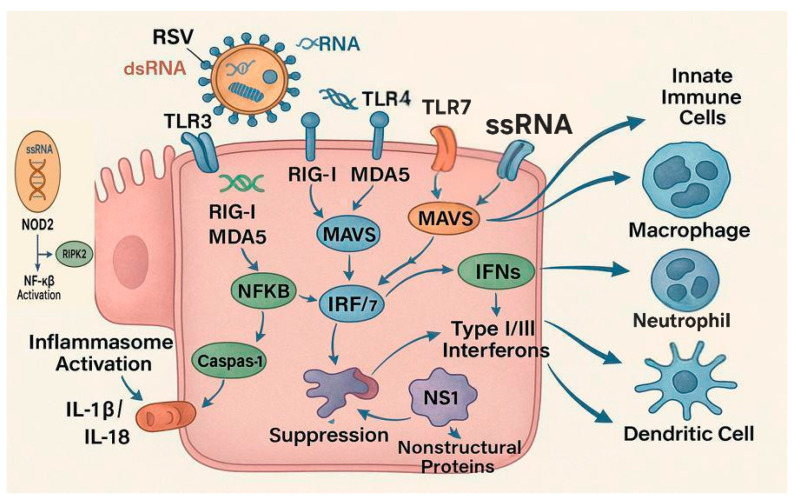
Schematic of innate immune responses to respiratory syncytial virus (RSV). Viral RNA and proteins are recognized by Toll-like receptors (TLRs), RIG-I-like receptors (RLRs), and cytosolic sensors such as NOD2 and the NLRP3 inflammasome. These pathways induce type I and III interferons and proinflammatory cytokines. Recruitment and activation of innate immune cells—including dendritic cells, macrophages, neutrophils, natural killer (NK) cells, and group 2 innate lymphoid cells (ILC2s)—lead to viral control but may also contribute to lung injury and immunopathology. NOD2 and inflammasome activation are critical components of epithelial and monocytic antiviral responses, promoting chemokine-mediated leukocyte infiltration and airway inflammation.

Figure 2 illustrates the innate immune responses to RSV.

#### 2.1.4. Suppression of Interferon Signaling

RSV employs its nonstructural proteins, NS1 and NS2, to suppress the host’s IFN response, a central component of innate antiviral defense. NS1 has been shown to inhibit the activation of interferon-stimulated response elements (ISRE) and gamma-activated sequence (GAS) promoters, resulting in decreased expression of ISGs, including MxA, Ubiquitin-Specific Peptidase 18 (USP18), and ISG15. This suppression is mediated through interference with the Janus kinase–signal transducer and activator of transcription (JAK/STAT) signaling cascade. Specifically, NS1 impairs STAT1 nuclear translocation by blocking its interaction with the import adapter Karyopherin Subunit Alpha 1 (KPNA1), thereby preventing the transcriptional induction of ISGs downstream of type I IFN receptors [36].

In addition, both NS1 and NS2 facilitate the degradation of STAT2, a key transcription factor in type I IFN signaling [37]. The removal of STAT2 by proteasomal degradation severely limits IFN-α/β responsiveness in infected cells and allows for unchecked viral replication during early infection. This degradation is especially effective in airway epithelial cells, where interferon responses are typically robust.

Together, these mechanisms enable RSV to establish early replication niches by blunting the antiviral state normally induced by IFNs. These findings underscore the importance of NS1 and NS2 as key viral immune evasion factors and potential targets for host-directed antiviral strategies [17,36].

### 2.2. Adaptive Immune Response

The adaptive immune response to RSV is critical for viral clearance and long-term immune memory but is also implicated in disease severity and immunopathology. Following innate immune activation, antigen-presenting cells, especially dendritic cells, migrate to regional lymph nodes where they activate naïve T and B lymphocytes, initiating the adaptive phase of the immune response.

#### T and B Cell Dynamics in RSV Infection: Protective Immunity and Pathogenic Skewing

CD8+ cytotoxic T lymphocytes (CTLs) are essential for clearing RSV-infected cells, particularly in the lungs. These cells recognize endogenously processed RSV-derived peptides presented on major histocompatibility complex (MHC) class I molecules. The viral peptides originate from newly synthesized viral proteins within infected epithelial cells, which are degraded by the proteasome and transported into the endoplasmic reticulum via the transporter associated with antigen processing (TAP) complex. Once loaded onto MHC class I molecules, these peptides are displayed on the cell surface, where they are recognized by CD8+ cytotoxic T lymphocytes. Upon activation, these lymphocytes release perforin and granzyme B to induce apoptosis and secrete IFN-γ to amplify the antiviral response [38]. However, excessive or poorly regulated CD8+ responses can also contribute to tissue damage and airway dysfunction, particularly during primary infection in neonates [39].

CD4+ T helper cells are activated through a three-signal process involving recognition of RSV-derived peptides presented on MHC class II molecules, co-stimulatory interactions (e.g., CD28–CD80/86), and cytokine-driven differentiation cues. CD4+ T helper cells orchestrate both cellular and humoral immune responses. RSV-specific CD4+ T cells differentiate into various subsets, including Th1, Th2, Th17, and regulatory T cells (Tregs), depending on the cytokine environment and co-stimulatory signals. Th1 cells promote viral clearance via IFN-γ and IL-2 production, while Th2-skewed responses—characterized by IL-4, IL-5, and IL-13 secretion—are associated with allergic-type inflammation, eosinophilia, mucus overproduction, and enhanced disease [9,40].

The Th17 response, marked by IL-17A and IL-22 production, also contributes to inflammation, neutrophil recruitment, and epithelial barrier modulation in RSV infection. While beneficial for mucosal defense, Th17 activity may exacerbate immunopathology in severe disease [41].

Tregs expressing Forkhead box P3 (FOXP3) are critical for limiting immune-mediated lung injury. RSV infection induces Treg expansion, which may prevent excessive inflammation; however, in severe disease, Treg function may be impaired or insufficient. RSV infection induces the expansion of Treg through mechanisms involving tolerogenic dendritic cell programming, RSV protein-mediated immune modulation, and IL-2-driven proliferation of natural Tregs. RSV-infected DCs secrete TGF-β and IL-10, promoting Foxp3+ Treg differentiation, while the RSV G protein interferes with dendritic activation via CX3CR1 engagement. Although Tregs help limit immunopathology by dampening excessive inflammation, their expansion in severe RSV cases may suppress protective Th1 and cytotoxic responses, contributing to impaired viral clearance [42].

The balance between effector T cells and regulatory T cells (Tregs) is a critical determinant of RSV disease outcome. Effective antiviral defense requires robust effector T cell responses, particularly Th1 and cytotoxic CD8^+^ T cells, to eliminate infected cells. However, unrestrained effector activity may lead to excessive cytokine release, neutrophilic inflammation, and airway damage. Tregs serve to restrain this inflammation, preserving tissue integrity. When this balance is disrupted—either by effector cell dominance or excessive Treg activity—disease severity increases due to either immunopathology or viral persistence, respectively. Thus, immune homeostasis between effector and regulatory arms is essential for the optimal resolution of RSV infection [41,43,44]

The outcome of RSV infection is critically influenced by the quality and polarization of the T cell response. While effective CD4+ and CD8+ T cell responses are required for viral clearance, RSV is known to induce dysregulated T cell immunity, leading to inadequate viral control or excessive inflammation.

A hallmark of RSV infection, particularly in infants, is the skewing of CD4+ T cells toward a Th2 phenotype, characterized by the overproduction of IL-4, IL-5, and IL-13. This Th2-biased response is associated with eosinophilic inflammation, mucus hypersecretion, and enhanced disease severity [45]. The phenomenon was classically illustrated in infants who received a formalin-inactivated RSV vaccine and subsequently developed vaccine-enhanced disease, marked by severe Th2-mediated pathology upon natural infection [46].

In addition to Th2 dominance, Th17 responses, defined by IL-17A and IL-22 production, are also upregulated in severe RSV cases. While IL-17 can enhance mucosal defense and neutrophil recruitment, uncontrolled Th17 activity contributes to airway hyperreactivity and epithelial damage [47]. The combination of Th2 and Th17 cytokines forms a non-protective inflammatory milieu that exacerbates lung injury without effectively clearing the virus.

Simultaneously, RSV manipulates T cell function via immune checkpoint pathways. Infected antigen-presenting cells upregulate PD-L1, which interacts with PD-1 on activated T cells, leading to T cell exhaustion, reduced proliferation, and diminished cytokine secretion [48]. These exhausted T cells exhibit impaired cytotoxicity and memory formation, especially in the CD8+ compartment.

Furthermore, RSV disrupts the balance between effector T cells and Tregs. While Tregs expressing FOXP3 are expanded during infection to suppress excessive inflammation, their suppressive capacity may be inadequate or transient, especially in infants [42]. This imbalance permits both ineffective viral clearance and unchecked inflammation, further complicating disease resolution.

B lymphocytes are activated via interaction with follicular helper T cells (Tfh) and differentiate into plasma cells that produce RSV-specific antibodies. Both mucosal IgA and serum IgG play roles in neutralizing the virus and limiting spread. Neutralizing antibodies target key epitopes on the F and attachment (G) glycoproteins of RSV. The most potent neutralizing antibodies against RSV target conformational epitopes on the prefusion F glycoprotein, particularly antigenic site Ø, which lies at the apex of the trimer and is unique to the prefusion conformation. Additional neutralizing activity derives from antibodies against site II (recognized by palivizumab), site IV, and site III. The G glycoprotein is a secondary target, with some neutralizing antibodies recognizing its central conserved domain, which includes the immunomodulatory CX3C motif [48,49]. High titers of maternal antibodies transferred via the placenta or provided passively are associated with protection in early infancy [50]. More specifically, high titers of RSV-specific IgG antibodies are transferred transplacentally during the third trimester via FcRn-mediated mechanisms, providing infants with passive systemic protection in early life. Studies have shown a strong correlation between maternal and cord blood neutralizing antibody titers, with higher maternal levels conferring reduced risk of RSV-related hospitalization in infants [51]. In addition, secretory IgA antibodies present in breast milk may offer mucosal protection against RSV infection in the respiratory tract, although their protective efficacy is more limited and localized [52].

However, RSV has developed mechanisms to impair humoral immunity. The RSV G glycoprotein exhibits substantial structural variability and extensive glycosylation, which enable it to evade immune detection. It also functions as a decoy by binding neutralizing antibodies and diverting them away from more conserved viral targets, thereby impairing effective antibody-mediated neutralization [53].

Furthermore, RSV infection can lead to delayed or weak antibody affinity maturation and memory B cell formation, contributing to susceptibility to reinfection [54].

In addition to direct cytolytic activity, RSV-specific CD8+ T cells exhibit phenotypic diversity depending on the stage of infection and tissue localization. In murine models, lung-resident memory CD8+ T cells (Trm) persist after RSV clearance and confer rapid local protection upon reinfection [55]. However, during acute infection, CD8+ T cells may adopt an exhausted phenotype—characterized by upregulation of PD-1, LAG-3, and TIM-3—which impairs their antiviral efficacy, particularly in neonates or in the setting of high viral load [56]. Moreover, the kinetics of CD8+ infiltration versus viral replication may influence disease severity; a delayed but intense CD8+ influx can promote immunopathology rather than protection [57].

Among CD4+ T cell subsets, follicular helper T cells (Tfh) are essential for optimal B cell maturation in germinal centers. These cells express CXCR5 and Bcl-6 and secrete IL-21, driving somatic hypermutation, affinity maturation, and the generation of long-lived plasma cells and memory B cells [58]. RSV infection has been shown to impair Tfh responses and germinal center formation, particularly in infants, resulting in suboptimal humoral memory and predisposition to reinfection [59]. The skewing of CD4+ T cell differentiation toward Th2 rather than Tfh in early life may be one mechanism behind the poor durability of RSV-specific antibody responses.

Although RSV induces the formation of virus-specific B cells, the longevity and functionality of these responses remain suboptimal. Memory B cells generated during natural RSV infection often display limited reactivation capacity and reduced affinity maturation upon re-exposure [60]. Studies have shown that RSV can inhibit germinal center reactions via IL-21 suppression and defective Tfh signaling, impairing the quality of long-lived plasma cell and memory B cell pools [61]. This dysfunctional humoral memory may explain the frequent reinfections throughout life, even in the presence of detectable RSV-specific antibodies.

Secretory IgA plays a crucial role in mucosal immunity against RSV, neutralizing the virus at the entry point and preventing epithelial cell infection. However, RSV-specific IgA responses are often short-lived and poorly maintained in the absence of repeated exposure [4]. Furthermore, RSV can disrupt local immune architecture by interfering with the function of antigen-presenting cells in mucosa-associated lymphoid tissue (MALT), leading to impaired class-switch recombination and IgA production. The failure to generate durable mucosal IgA responses contributes to the limited sterilizing immunity following natural RSV infection.

The humoral immune response plays a pivotal role in neutralizing RSV and preventing reinfection. However, RSV has evolved several strategies to impair B cell function, evade neutralizing antibodies, and limit long-term protective immunity.

A central mechanism of humoral evasion is the secretion of the soluble form of the G glycoprotein. This secreted decoy mimics the membrane-bound G protein and binds circulating neutralizing antibodies, thereby reducing their availability to engage infectious virions [62]. In addition, the G protein’s CX3C motif, which structurally mimics the host chemokine fractalkine, interferes with immune cell trafficking and modulates the local cytokine environment, further dampening effective B cell activation [63].

RSV also limits germinal center (GC) formation in secondary lymphoid tissues. It has been shown that RSV-infected mice and human infants exhibit impaired GC responses, with reduced numbers of Tfh cells and suboptimal B cell proliferation, affinity maturation, and class-switch recombination [64]. As a result, the antibody repertoire generated during primary infection is often of low affinity, short-lived, and skewed toward non-neutralizing epitopes.

Moreover, RSV infection interferes with memory B cell generation. Even in previously infected individuals, reinfections are common, and the neutralizing antibody titers wane rapidly. RSV can induce the apoptosis of activated B cells and reduce the survival signals necessary for memory cell maintenance, such as IL-21 and B-cell activating factor (BAFF) [4]. These deficiencies explain the lack of sterilizing immunity after natural infection and complicate vaccine design.

Finally, RSV has been shown to modulate the function of plasmablasts and antibody-secreting cells, altering their trafficking patterns and cytokine responsiveness. In severe cases, this contributes not only to insufficient viral neutralization but also to persistent inflammation mediated by immune complexes and complement activation.

Altogether, these strategies enable RSV to escape antibody-mediated immunity and establish recurrent infections, underscoring the need for interventions that can preserve or restore effective B cell responses.

### 2.3. Modulation of Pattern Recognition Receptors (PRRs)

RSV has evolved mechanisms to interfere with host PRRs, which are crucial components of the innate immune system responsible for detecting viral components and initiating antiviral responses. Key PRRs involved in RSV recognition include TLRs and RIG-I-like receptors (RLRs), such as RIG-I and MDA5.

#### 2.3.1. Toll-like Receptors (TLRs)

TLRs, particularly TLR3 and TLR4, play significant roles in recognizing RSV. TLR3 detects double-stranded RNA, a replication intermediate of RSV, while TLR4 recognizes the RSV F protein. Activation of these receptors leads to the production of type I interferons and pro-inflammatory cytokines. However, RSV can modulate TLR signaling pathways to dampen the immune response. For instance, the RSV G protein has been shown to interfere with TLR4 signaling, reducing the production of pro-inflammatory cytokines and type I IFNs, thereby facilitating viral persistence [65,66].

#### 2.3.2. RIG-I-like Receptors (RLRs): RIG-I and MDA5

RLRs, including RIG-I and MDA5, are cytoplasmic sensors that detect viral RNA. RIG-I primarily recognizes short double-stranded RNA with 5’-triphosphate ends, while MDA5 detects long double-stranded RNA. Upon recognition, these receptors initiate signaling cascades, leading to the production of type I IFNs. RSV can inhibit RLR signaling pathways, thereby suppressing the host’s antiviral responses. For example, RSV nonstructural proteins NS1 and NS2 can interfere with the activation of RIG-I and MDA5, leading to reduced IFN production [67,68,69].

### 2.4. Glycoprotein G and CX3CR1 Interaction

The RSV G protein contains a CX3C chemokine motif that mimics the host chemokine fractalkine (CX3CL1), allowing it to bind to the CX3CR1 receptor on immune cells. This interaction can impair the migration and function of CX3CR1-expressing immune cells, such as cytotoxic T lymphocytes and natural killer cells, thereby modulating the immune response to RSV infection [70,71]. The mimicry of host proteins by viral components is a well-recognized mechanism by which viruses evade immunity and may contribute to immune dysregulation. In the case of RSV, binding of the G protein to CX3CR1 has been shown to suppress antiviral Th1-type responses and promote a Th2-skewed immune profile, thereby impairing viral clearance and exacerbating inflammation [9,72]. To date, there is no direct evidence linking RSV infection to classical autoimmune diseases; however, the virus’s ability to mimic host chemokines and modulate immune pathways raises theoretical concerns about its potential to contribute to immune dysregulation [9,61,72].

### 2.5. Inhibition of Dendritic Cell Function

DCs are central to the orchestration of antiviral immunity, acting as key antigen-presenting cells (APCs) that bridge innate and adaptive immune responses. Upon RSV infection, dendritic cells are recruited to the respiratory mucosa, where they capture viral antigens and migrate to draining lymph nodes to prime naïve T cells. However, RSV actively interferes with the function, phenotype, and migration of DCs to suppress effective immune activation [48,73].

RSV-infected DCs display impaired maturation, characterized by the decreased expression of co-stimulatory molecules such as CD80, CD86, and CD83, as well as reduced MHC class II expression [23]. This immature phenotype leads to inadequate T cell priming and a suboptimal adaptive immune response. Additionally, RSV infection reduces the ability of DCs to secrete IL-12, a cytokine essential for polarizing T cells toward a Th1 antiviral phenotype [44]. This contributes to the characteristic Th2 bias observed in RSV infection, which is associated with enhanced pathology.

Mechanistically, RSV can alter the expression of C-C chemokine receptor type 7 (CCR7), which is the chemokine receptor that governs DC migration from peripheral tissues to secondary lymphoid organs. Downregulation of CCR7 expression or altered responsiveness to its ligands [C-C motif chemokine ligand 19 (CCL19) and C-C motif chemokine ligand 21 (CCL21)] results in impaired trafficking of infected DCs to lymph nodes, delaying or weakening the initiation of the adaptive response [74].

Moreover, RSV-infected DCs may exhibit enhanced expression of programmed death-ligand 1 (PD-L1), an immune checkpoint molecule that engages programmed cell death protein 1 (PD-1) on T cells and contributes to T cell exhaustion and reduced cytokine production [75]. This further limits T cell proliferation and effector function, allowing viral persistence.

The impairment of DC function represents a crucial immune evasion mechanism, particularly in infants and neonates, whose DCs already exhibit reduced responsiveness due to developmental immaturity. RSV thus exploits both viral and host-derived factors to attenuate DC-mediated immunity, facilitating reinfection and prolonged inflammation.

## 3. Immunopathology and Host Damage

**Figure 3:**
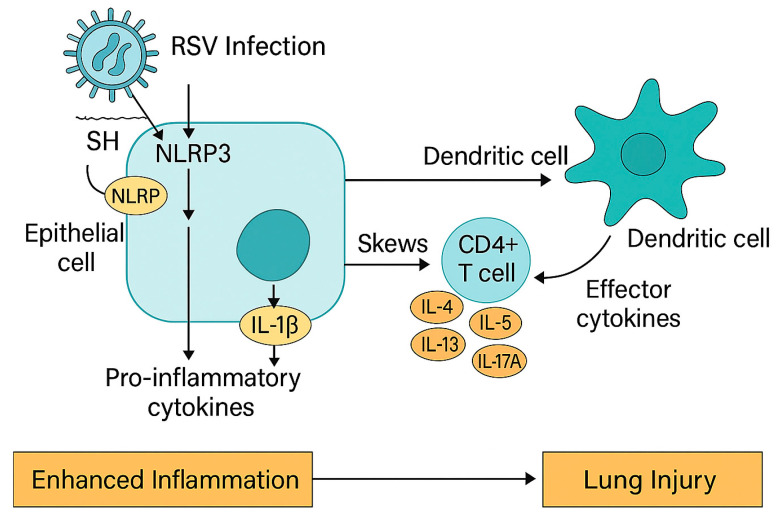
Molecular mechanism of RSV-induced inflammation via NLRP3 inflammasome activation. Respiratory syncytial virus (RSV) infects airway epithelial cells, triggering activation of the NLRP3 inflammasome via its SH protein. This leads to the cleavage and secretion of interleukin-1β (IL-1β), a potent pro-inflammatory cytokine. IL-1β promotes recruitment of dendritic cells (DCs), which prime naïve CD4+ T cells. The inflammatory milieu skews CD4+ T cells toward Th2 and Th17 phenotypes, producing effector cytokines, including IL-4, IL-5, IL-13, and IL-17A. These cytokines contribute to eosinophilic inflammation, mucus hypersecretion, and neutrophil recruitment, ultimately resulting in lung injury. This figure does not include interferon suppression, CD8+ T cell responses, or regulatory mechanisms involved in RSV pathogenesis.

RSV-induced lung injury results from both viral replication and immune-mediated damage through a variety of mechanisms (Figure 3).

### 3.1. Role of the Immune Response in Lung Injury

While the immune system plays a vital role in the clearance of RSV, dysregulated or excessive immune activation can lead to significant pulmonary injury, particularly in infants and elderly patients. RSV-associated bronchiolitis and pneumonia are frequently not the result of uncontrolled viral replication alone but are also driven by host-derived immunopathological processes.

A key contributor to lung damage is the hyperactivation of innate immune cells, particularly neutrophils, which dominate the cellular infiltrates in the lower respiratory tract during acute RSV infection [76]. Although neutrophils assist in viral clearance, they also release reactive oxygen species (ROS), proteolytic enzymes, and NETs, which can damage the airway epithelium, promote mucus hypersecretion, and impair gas exchange [77].

In parallel, monocytes and alveolar macrophages secrete large amounts of pro-inflammatory cytokines such as TNF-α, IL-6, and IL-1β, which amplify local inflammation and contribute to vascular leakage, edema, and airway obstruction [20]. These cytokines also upregulate adhesion molecules on endothelial cells, promoting further immune cell infiltration and worsening the inflammatory cycle.

The adaptive immune response, especially Th2- and Th17-skewed CD4+ T cell responses, contributes to chronic inflammation and tissue remodeling. Th2 cytokines such as IL-4, IL-5, and IL-13 enhance eosinophilic inflammation and mucus production, while IL-17 from Th17 cells promotes neutrophil recruitment and epithelial barrier dysfunction [41]. These responses can be protective under tightly regulated conditions but become pathogenic when exaggerated or prolonged.

Histopathological studies of severe RSV cases reveal extensive airway epithelial sloughing, submucosal edema, peribronchiolar lymphocytic infiltration, and mucus plugging, which are features that correlate more strongly with the magnitude of the host immune response than with viral load [11,78,79].

Notably, the age-dependent nature of immunopathology is a hallmark of RSV disease. Neonates and infants exhibit heightened Th2 bias and lower IFN responses, while older adults may experience immune senescence and delayed clearance, both of which predispose to lung injury via different mechanisms [80].

### 3.2. Chronic Sequelae and Long-Term Impact of Immunopathology

Beyond acute bronchiolitis and pneumonia, RSV infection—particularly during early life—has been associated with long-term respiratory sequelae, including recurrent wheezing, asthma-like symptoms, and reduced pulmonary function. These outcomes are increasingly understood as consequences of immune-mediated injury and dysregulated repair mechanisms initiated during the acute phase of infection.

Epidemiological studies have shown that severe RSV bronchiolitis in infancy is a strong predictor of recurrent wheezing and asthma in childhood and adolescence [80]. The underlying pathophysiology appears to involve persistent airway remodeling, initiated by immune-mediated epithelial damage, subepithelial fibrosis, and goblet cell hyperplasia during acute infection [81]. These structural changes are driven by chronic Th2- and Th17-type inflammation, including the sustained activity of cytokines such as IL-13, IL-17A, and TGF-β, which promote mucus production and fibrosis.

The presence of neutrophil elastase, matrix metalloproteinases, and oxidative stress products in RSV-infected lungs contributes to extracellular matrix degradation and abnormal repair, predisposing the lung to increased airway hyperreactivity and reduced compliance [24]. In addition, RSV infection may affect the epithelial–mesenchymal trophic unit, further altering developmental trajectories of the airways in infants [82].

Furthermore, the immunological imprinting that occurs during RSV infection may lead to long-lived alterations in both the innate and adaptive immune compartments. For example, infants who experience severe RSV may develop memory T cell populations skewed toward Th2, which can be reactivated upon later exposures to RSV or other allergens, promoting asthma-like pathology [61]. These immune imprints may also impair the host’s capacity to respond appropriately to subsequent respiratory infections.

It remains debated whether RSV plays a causal role in asthma development or simply unearths a pre-existing predisposition. However, animal models and human cohort studies support a mechanistic link between early-life RSV immunopathology and later airway disease, particularly in genetically or immunologically susceptible individuals [83,84].

### 3.3. Age-Dependent Differences in Immunopathology

The immunopathological manifestations of RSV infection vary significantly across the lifespan, with the most severe clinical outcomes seen in infants and older adults. These age-dependent differences reflect fundamental variations in immune system development, function, and regulation, which in turn shape the host’s capacity to respond to RSV infection and repair tissue injury.

#### 3.3.1. Infants and Young Children

In neonates and infants, the immune system is functionally immature. RSV infection in this age group is characterized by a predominant Th2-type immune response, reduced type I interferon production, and impaired cytotoxic T cell activity [8]. These deficiencies contribute to inadequate viral clearance and excessive inflammation. In particular, alveolar macrophages and dendritic cells in neonates exhibit lower expression of co-stimulatory molecules and cytokines such as IL-12, leading to poor CD8+ T cell priming [85,86].

Moreover, neonates have a limited pool of tissue-resident memory T cells and a bias toward regulatory and tolerogenic immune pathways, likely an evolutionary adaptation to limit inflammation during early development. While this protects against overwhelming immune responses, it renders neonates more susceptible to viral replication and immunopathology mediated by non-protective inflammation, particularly involving Th2 and Th17 cytokines [87].

The narrow airway caliber, high compliance of lung tissue, and relatively high neutrophil-to-lymphocyte ratios in infants also contribute mechanically and immunologically to the severity of bronchiolitis and airway obstruction [88].

#### 3.3.2. Older Adults

In contrast, older adults experience immune senescence, which affects both innate and adaptive immune compartments. Key features include reduced function of natural killer (NK) cells, dendritic cells, and CD8+ effector memory T cells, as well as impaired interferon responses. These changes result in delayed viral clearance, higher viral loads, and prolonged inflammation [89].

At the same time, chronic low-grade inflammation—commonly referred to as “inflammaging”—predisposes older adults to exaggerated responses to RSV, including cytokine overproduction and tissue damage, particularly in those with comorbidities like COPD or cardiovascular disease [90]. The recruitment of neutrophils and monocytes may be less efficient but more damaging due to the impaired resolution of inflammation.

Furthermore, antibody responses in older adults are typically weaker and less durable, with limited isotype switching and affinity maturation, compromising protection despite prior exposures [91].

Neutralizing antibody titers in this population tend to be lower at baseline and demonstrate attenuated responses following both natural infection and immunization. In the RENOIR trial, administration of a bivalent RSV prefusion F vaccine led to robust increases in neutralizing titers against RSV-A and RSV-B (geometric mean fold-rise: 12.3 and 10.2, respectively); however, titers waned significantly over six months, highlighting the need for durable boosting strategies [14]. Similarly, in the CYPRESS study evaluating the Ad26.RSV.preF vaccine, antibody responses peaked at day 15 post-vaccination but declined by day 90, despite initial increases in RSV-A and RSV-B GMTs (12-fold and 9-fold, respectively) [13]. The ARESVI-006 trial, assessing the efficacy of the RSVPreF3 OA vaccine, also reported peak neutralizing GMT increases of 11-fold for RSV-A and 9.8-fold for RSV-B, with titers decreasing by approximately 50% by month 6 [15]. These patterns are consistent with immunosenescence-driven limitations in maintaining long-lived humoral immunity. Reduced antibody avidity, suboptimal germinal center responses, and impaired memory B cell formation in older adults further impair the generation of high-affinity, long-lasting protective antibodies. Together, these findings underscore the importance of designing vaccine strategies that either induce sustained antibody titers or incorporate periodic re-immunization in elderly populations at risk of severe RSV disease.

#### 3.3.3. Implications

These contrasting immunopathological profiles in infants versus the elderly underscore the importance of age-specific approaches to RSV prevention and treatment. Strategies such as maternal immunization, monoclonal antibody prophylaxis for infants, and vaccination of older adults must consider the distinct immunological vulnerabilities of each population [92].

### 3.4. Overproduction of Cytokines: Linking Immunopathology to Bronchiolitis and Airway Remodeling

One of the defining features of severe RSV infection is the excessive production of pro-inflammatory cytokines and chemokines. While these mediators are essential for antiviral defense and leukocyte recruitment, their dysregulated release contributes to bronchiolitis, tissue injury, and long-term airway remodeling. RSV-infected airway epithelial cells, macrophages, and dendritic cells produce elevated levels of cytokines such as IL-6, IL-1β, TNF-α, and granulocyte-macrophage colony-stimulating factor (GM-CSF), along with chemokines, including IL-8, macrophage inflammatory protein 1-alpha (MIP-1α), CCL5, and CXCL10 [93,94,95]. These molecules orchestrate a robust recruitment of neutrophils, eosinophils, monocytes, and T lymphocytes into the airways.

Among these, IL-8 plays a pivotal role in neutrophil chemotaxis, and its elevated expression has been linked to the dense neutrophilic infiltrates observed in the bronchoalveolar lavage fluid of infants with RSV bronchiolitis [25]. Although neutrophils are critical for limiting viral dissemination, their release of granule contents such as elastase and myeloperoxidase, as well as the formation of NETs, exacerbates epithelial injury and stimulates mucus hypersecretion [25]. IL-6, a multifunctional cytokine, further amplifies inflammation by promoting acute-phase protein production, enhancing Th17 differentiation, and supporting B cell survival. Importantly, persistently high IL-6 levels have been associated with increased disease severity, prolonged hospitalization, and the need for respiratory support in RSV-infected infants [95].

The downstream effects of this pro-inflammatory microenvironment include the development of bronchiolitis, which is histologically characterized by epithelial sloughing, mucus plugging, submucosal edema, and small airway obstruction. These changes correlate more strongly with host immune activation than with viral load, indicating a key role for immunopathology in disease progression [96]. Furthermore, cytokine-mediated disruption of epithelial barrier integrity facilitates viral spread to deeper lung tissues and sustains the cycle of inflammation and injury [97].

Prolonged inflammation in severe RSV cases leads to airway remodeling, a process that encompasses subepithelial fibrosis, goblet cell hyperplasia, and smooth muscle hypertrophy. IL-13 and IL-17, which are downstream products of Th2 and Th17 polarization respectively, contribute to mucus metaplasia, collagen deposition, and thickening of the airway wall [98,99]. These structural changes particularly concern infants, as they have been implicated in the development of chronic wheezing and asthma later in life, especially among those hospitalized with severe RSV bronchiolitis [100].

Taken together, these findings highlight that the overproduction of cytokines represents a critical link between acute RSV infection and both short-term respiratory dysfunction and long-term airway disease. Targeting specific inflammatory pathways—such as those mediated by IL-6, IL-8, IL-13, or IL-17—may offer future therapeutic strategies to reduce immunopathology while preserving effective antiviral immunity.

## 4. Monoclonal Antibodies and Vaccines

Monoclonal antibodies (mAbs) represent a crucial tool in the prevention of RSV infection, particularly in vulnerable pediatric populations. While active immunization through vaccines stimulates the host immune system to generate long-term protection, it may not be feasible or effective in certain high-risk groups, such as neonates, young infants, or immunocompromised individuals, whose immune responses are either immature or suppressed [100,101]. In such cases, passive immunization with monoclonal antibodies offers immediate, albeit temporary, protection by directly neutralizing the virus [102]. The use of monoclonal antibodies is particularly warranted in infants at high risk for severe RSV disease, including those born prematurely, those with chronic lung or congenital heart disease, and those with significant immunodeficiencies [103]. This approach reduces RSV-associated morbidity, hospitalizations, and the burden on healthcare systems during seasonal outbreaks [102].

### 4.1. Palivizumab

Palivizumab is a humanized IgG1 monoclonal antibody that targets the A antigenic site of the RSV F protein, thereby inhibiting viral entry into host cells. It is administered intramuscularly monthly during RSV season and is approved for high-risk infants, including those born prematurely, with bronchopulmonary dysplasia, or with hemodynamically significant congenital heart disease [104].

The pivotal IMpact-RSV trial demonstrated that palivizumab prophylaxis reduced RSV-related hospitalizations by 55% in high-risk infants (10.6% in the placebo group vs. 4.8% in the palivizumab group; *p* < 0.001) [102]. Subsequent studies have corroborated its efficacy, reporting reductions in RSV-related hospitalizations ranging from 45% to 82% in various high-risk pediatric populations [105].

Despite its demonstrated efficacy, the widespread use of palivizumab is limited by factors such as high cost, the need for multiple doses, and limited applicability to broader infant populations [106].

### 4.2. Nirsevimab

Nirsevimab is a long-acting monoclonal antibody designed to provide passive immunity against RSV by targeting a conserved epitope on the prefusion conformation of the F protein. It incorporates YTE (M252Y/S254T/T256E) Fc modifications to extend its half-life, allowing for a single intramuscular dose to offer protection throughout the RSV season [107].

In the phase 3 MELODY trial, a single dose of nirsevimab reduced the incidence of medically attended RSV-associated LRTIs by 74.5% compared to the placebo in healthy late-preterm and term infants during their first RSV season [107]. Furthermore, a pooled analysis of two randomized, placebo-controlled studies showed that prophylaxis with nirsevimab had an efficacy of 79.5% against medically attended RSV LRTIs [108].

Real-world data have further supported these findings. A study published in the New England Journal of Medicine reported that nirsevimab was 83.2% effective in preventing hospitalization and 75.7% effective in reducing severe RSV cases among infants under one year of age [109].

The U.S. Food and Drug Administration approved nirsevimab (marketed as Beyfortus) in July 2023 for the prevention of RSV lower respiratory tract disease in neonates and infants entering their first RSV season. Its single-dose regimen and broader efficacy profile position nirsevimab as a significant advancement in RSV prophylaxis, potentially offering a more practical and effective alternative to palivizumab [110].

**Figure 4:**
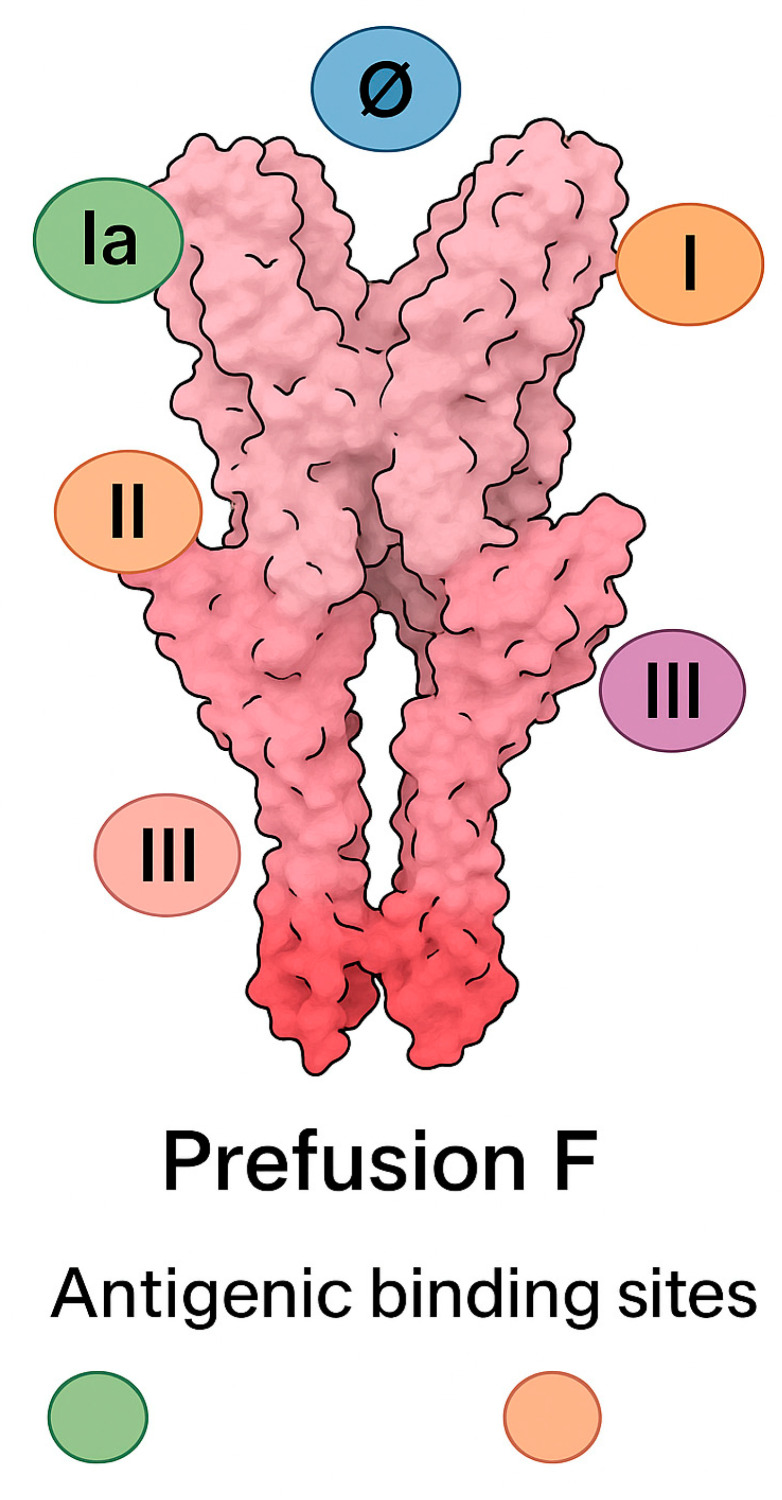
Structure of the RSV prefusion F protein and monoclonal antibody binding sites. This model illustrates the trimeric prefusion conformation of the RSV F glycoprotein, highlighting key antigenic sites (Ø, Ia, I, II, III) recognized by monoclonal antibodies. Site Ø is a conformational epitope exclusive to the prefusion state and the primary binding site for highly potent antibodies such as nirsevimab. Site II, shared between pre- and post-fusion states, is the binding site of palivizumab. Mapping these epitopes is critical for the rational design of next-generation RSV vaccines and therapeutics.

A detailed understanding of the antigenic landscape of the RSV prefusion F protein has been essential for the development of high-potency monoclonal antibodies. Site Ø, in particular, is a major target of neutralizing antibodies such as nirsevimab, which binds with high affinity to this prefusion-specific epitope (Figure 4).

### 4.3. Vaccines

Several RSV vaccines have now been approved for use in specific age groups, representing a milestone in RSV prevention. The RSVPreF3 and RSVpreF vaccines are licensed for adults ≥60 years of age and are based on stabilized prefusion F protein platforms. These vaccines elicit robust neutralizing antibody responses in older adults, although waning immunity and reduced cell-mediated responses remain challenging due to immunosenescence [14,15]. In contrast, infants and young children face unique immunologic barriers, including an immature adaptive immune system, reduced interferon responses, and interference by maternal antibodies [8,111].

While maternal immunization with RSVpreF is now recommended during the third trimester to provide passive protection to infants during the first months of life, direct immunization of infants remains under development. One promising approach, the mRNA-based vaccine mRNA-1345, has shown potent immunogenicity and efficacy in adults; however, emerging data raise safety concerns when considering infant immunization, particularly in relation to febrile reactions and local reactogenicity [112]. These findings underscore the need for caution in extending mRNA platforms to pediatric populations.

Future RSV prevention strategies will likely require age-tailored approaches, such as protein-based vaccines or maternal immunization for infants, high-dose or adjuvanted formulations for older adults, and potentially live-attenuated or vectored vaccines for children. Immunologic challenges such as T cell immaturity in infants and immunosenescence in older adults must inform vaccine design, delivery schedules, and long-term boosting strategies.

## 5. Cytokine Modulation and Anti-Inflammatory Agents

RSV infection often leads to an excessive inflammatory response, with marked overproduction of cytokines such as IL-6, IL-8, TNF-α, and IL-1β, which contribute significantly to lung injury and disease severity. Therapeutic modulation of this cytokine cascade has emerged as a potential approach to limit tissue damage and reduce RSV-related morbidity.

### 5.1. Corticosteroids

Corticosteroids are broad-spectrum anti-inflammatory agents that exert their effects by binding to cytoplasmic glucocorticoid receptors (GRs), which then translocate to the nucleus and interact with glucocorticoid response elements (GREs) on DNA. This leads to the suppression of the transcription of pro-inflammatory cytokines (e.g., IL-1β, TNF-α, IL-6) and the upregulation of anti-inflammatory mediators such as IL-10 and annexin A1 [113]. Despite these mechanisms, clinical trials of corticosteroids in RSV bronchiolitis have produced inconsistent results. A Cochrane meta-analysis concluded that corticosteroids, including dexamethasone and prednisolone, did not significantly reduce the risk of hospital admission, duration of hospitalization, or severity of illness in most infants with RSV [114].

Inhaled dexamethasone may reduce the length of hospitalization among infants with acute RSV bronchiolitis, especially among those born prematurely [115]. Nevertheless, corticosteroids may delay viral clearance, impair mucosal immunity, and increase the risk of secondary infections, raising concerns about their routine use in RSV [116]. For this reason, corticosteroids are not recommended as standard therapy in otherwise healthy infants with RSV bronchiolitis, though ongoing studies continue to explore their potential in high-risk populations.

### 5.2. IL-6 Blockade

IL-6 is a pleiotropic cytokine involved in fever, acute-phase protein synthesis, and B-cell differentiation. Its overexpression has been linked to more severe RSV infection, particularly in infants with prolonged hospitalization and greater oxygen requirement [117]. In other viral diseases, such as COVID-19, pharmacologic IL-6 blockade with tocilizumab (a monoclonal antibody against the IL-6 receptor) has been shown to reduce hyperinflammatory responses, mitigate lung injury, and improve clinical outcomes [118,119].

While tocilizumab has not yet been studied in RSV-infected patients, its success in dampening cytokine storms in COVID-19 provides a mechanistic rationale for its future evaluation in severe RSV cases. IL-6 blockade interrupts JAK-STAT signaling and suppresses downstream inflammatory gene transcription, potentially reducing pulmonary inflammation without compromising viral clearance [120]. Given that high serum IL-6 levels have been correlated with disease severity in children with RSV, IL-6 represents a promising target for immunomodulatory intervention [121].

### 5.3. JAK Inhibitors

The Janus kinase (JAK)–signal transducer and activator of transcription (STAT) pathway transmits intracellular signals from a range of cytokine receptors, including those for IL-2, IL-4, IL-6, and IFN-γ. Dysregulation of this pathway plays a central role in cytokine storm syndromes. JAK inhibitors, such as baricitinib and ruxolitinib, act by competitively inhibiting JAK1 and JAK2, thereby preventing STAT phosphorylation and nuclear translocation, which ultimately blunts the transcription of pro-inflammatory genes [122].

Although not yet evaluated in clinical RSV trials, preclinical studies have shown that JAK inhibition can significantly attenuate airway inflammation and reduce lung injury in models of viral pneumonia without abolishing essential antiviral responses [123]. Moreover, baricitinib has shown efficacy in pediatric inflammatory conditions such as hemophagocytic lymphohistiocytosis and COVID-19-associated MIS-C, supporting its potential utility in RSV-related hyperinflammation [124,125].

### 5.4. IL-1 and NLRP3 Inflammasome Inhibition

Activation of the NLRP3 inflammasome by RSV leads to cleavage of pro-IL-1β via caspase-1 and subsequent release of mature IL-1β, a central driver of neutrophilic inflammation and tissue injury. IL-1β promotes the chemotaxis of neutrophils and monocytes, enhances vascular permeability, and amplifies local cytokine production. Inhibition of this axis may therefore limit immunopathology in RSV infection [126].

Anakinra, an IL-1 receptor antagonist approved for rheumatoid arthritis and autoinflammatory syndromes, has been shown to reduce lung inflammation and improve outcomes in models of viral ARDS and hypercytokinemia [127]. Similarly, dapansutrile, a small-molecule inhibitor of NLRP3, has demonstrated anti-inflammatory activity in preclinical studies of influenza and SARS-CoV-2, and it may hold promise in RSV by reducing IL-1β production at the inflammasome level [128]. These approaches are under investigation in early-phase clinical trials targeting viral pneumonia, though specific RSV trials are still lacking [129].

## 6. Small Molecule Antivirals and Host-Directed Therapies

The therapeutic landscape for RSV has expanded beyond prophylactic monoclonal antibodies to include small molecule antivirals and host-directed therapies. These approaches aim to directly inhibit viral replication or modulate host cellular pathways to mitigate infection severity.

### 6.1. Direct-Acting Antivirals (DAAs)

#### 6.1.1. Ribavirin

Ribavirin, a guanosine analog, remains the only antiviral approved for RSV treatment. It inhibits viral RNA synthesis and capping. However, its clinical use is limited due to concerns about efficacy, toxicity, and cost [130].

#### 6.1.2. Presatovir (GS-5806)

Presatovir is an oral fusion inhibitor targeting the RSV F protein, preventing viral entry into host cells. In a randomized, double-blind, placebo-controlled human challenge trial, DeVincenzo et al. [131] evaluated the efficacy of GS-5806 (Presatovir)—an oral RSV entry inhibitor—in healthy adults experimentally infected with RSV. Participants were assigned to seven dosing cohorts and received GS-5806 or a placebo following confirmed RSV infection or five days post-inoculation. The study demonstrated that GS-5806 significantly reduced viral load (adjusted mean AUC: 250.7 vs. 757.7 log_10_ PFUe·h/mL; *p* < 0.001), total mucus production (6.9 g vs. 15.1 g; *p* = 0.03), and symptom scores (*p* = 0.005) compared to the placebo. Although some adverse events such as transient neutropenia and elevated alanine aminotransferase were more frequent in the treatment group, the drug was generally well tolerated. These findings indicate that GS-5806 can effectively reduce RSV replication and symptom severity in a controlled setting, supporting its potential as a therapeutic agent against RSV.

In a randomized, double-blind, placebo-controlled Phase 2b study [132] involving 60 hematopoietic cell transplant (HCT) recipients with confirmed RSV lower respiratory tract infection across 17 centers, participants received oral 200 mg of Presatovir or a placebo every four days for five total doses. The primary endpoint—the time-weighted average change in nasal RSV viral load through day 9—showed no significant difference between the Presatovir and placebo groups (−1.12 vs. −1.09 log_10_ copies/mL; *p* = 0.94). Secondary outcomes, including supplemental oxygen–free days, incidence of respiratory failure requiring mechanical ventilation, and all-cause mortality, also did not differ significantly between arms. Adverse event rates were similar in both groups (80% vs. 79%), and resistance-associated substitutions in the RSV fusion protein emerged in 6 of 29 Presatovir-treated patients. The trial concluded that, despite acceptable safety and tolerability, Presatovir did not confer clinical or virologic benefits in this immunocompromised population

#### 6.1.3. Lumicitabine (ALS-8176)

Lumicitabine, a nucleoside analog, inhibits the RSV RNA-dependent RNA polymerase. While early studies showed promise, subsequent trials revealed limited efficacy, leading to discontinuation of its development for RSV [133].

#### 6.1.4. Ziresovir (AK0529)

Ziresovir is a small molecule RSV fusion inhibitor. A phase 3 randomized, placebo-controlled trial evaluated ziresovir in 1–24-month-old children hospitalized with RSV infection, showing that a 5-day oral course significantly improved clinical symptoms (as measured by the Wang bronchiolitis score) by day 3 compared to the placebo. Ziresovir also led to a greater reduction in RSV viral load by day 5 (−2.5 vs. −1.9 log_10_ copies/mL), with particularly notable benefits in infants ≤6 months old and those with more severe disease. The treatment was generally well tolerated, with adverse events comparable to the placebo and no major safety concerns, supporting ziresovir’s potential as an effective antiviral therapy for RSV bronchiolitis in young children [134]. Another phase 3 double-blind trial evaluated ziresovir in infants aged ≤6 months hospitalized with confirmed RSV across 28 hospitals in China, showing that a 5-day oral course led to a significantly greater improvement in Wang bronchiolitis clinical scores by day 3 (−3.5 vs. −2.2; difference −1.2, *p* = 0.0004) compared to the placebo, with treatment-emergent drug-related adverse events occurring in 18% versus 11% but no serious drug-related events or deaths. Moreover, follow-up up to 4 February 2024 suggested durable benefit and a favorable safety profile, reinforcing ziresovir’s clinical potential in this vulnerable age group [135].

#### 6.1.5. Lonafarnib

Lonafarnib, a farnesyltransferase inhibitor, has demonstrated antiviral activity against RSV by disrupting prenylation processes essential for viral replication. Preclinical studies have shown its efficacy in inhibiting RSV replication in vitro [136].

### 6.2. Host-Directed Therapies

#### 6.2.1. Interferon Therapy

RSV evades host immunity in part by suppressing type I and III IFN responses. Administration of exogenous IFN-α or IFN-λ has been shown in vitro and in mouse models to reduce viral load and limit pathology [137]. However, their clinical utility is constrained by concerns over inflammation and delivery route, and no approved interferon-based therapies currently exist for RSV.

#### 6.2.2. Toll-like Receptor (TLR) Agonists and Antagonists

TLRs are essential sensors in the antiviral immune response. TLR7/8 agonists, such as imiquimod and resiquimod, activate plasmacytoid dendritic cells and enhance type I IFN production, which suppresses RSV replication in vitro [134]. Conversely, TLR4 antagonists have been proposed to limit excessive inflammation driven by RSV F protein–TLR4 interaction, especially in severe disease [138].

#### 6.2.3. Microbiome-Targeted Immunomodulation

The composition of the respiratory and gut microbiota significantly influences host responses to RSV. Infants with severe bronchiolitis often exhibit reduced abundance of commensal genera such as *Corynebacterium* and *Dolosigranulum* and increased presence of *Haemophilus* and *Streptococcus* [139]. These findings suggest that microbiome-modulating interventions, such as probiotics or microbial metabolite therapies, could potentially reduce disease severity and promote immune balance [140].

## 7. Challenges and Future Directions

### 7.1. Balancing Viral Clearance and Immunopathology

A core challenge in RSV immunotherapy is achieving a balance between effective viral clearance and minimizing immune-mediated lung damage. Robust immune activation is necessary to eliminate the virus, yet excessive cytokine production and cellular infiltration, including neutrophils, eosinophils, and T cells, can exacerbate bronchiolitis and contribute to long-term airway remodeling [92,141]. Recent reviews underscore that RSV immunopathology results from the following delicate equilibrium: too little response allows viral persistence, while too much triggers immunopathology [141,142]. Emerging host-directed immunotherapeutic strategies—such as targeted inhibitors of specific cytokines or modulation of innate signaling pathways—show promise in tailoring responses based on individual immune profiles, aiming to preserve antiviral immunity while limiting tissue damage [143].

### 7.2. Age- and Comorbidity-Specific Modulation

Immunopathology in RSV infection differs markedly across age groups and comorbid populations. In neonates and young infants, immune responses are skewed toward Th2 and Th17 polarization, accompanied by limited type I interferon production and immature cytotoxic responses, predisposing them to severe bronchiolitis and long-term sequelae [144,145]. In contrast, elderly individuals often exhibit immunosenescence, characterized by reduced T cell functionality, impaired memory responses, and a baseline of chronic low-grade inflammation, which together impair viral clearance and contribute to prolonged illness [146,147]. Additionally, individuals with underlying comorbidities—such as chronic lung disease, prematurity, or congenital heart disease—demonstrate increased susceptibility to severe RSV outcomes due to both structural and immunological vulnerabilities [148,149]. These distinct immunological profiles highlight the need for age-adapted immunotherapies and comorbidity-aware vaccine strategies that can optimize both efficacy and safety across diverse risk groups [150,151].

### 7.3. Long-Term Immunity and Reinfection

Natural RSV infection provides only partial and short-lived immunity, which allows reinfections to occur throughout life, even in individuals previously exposed. This limited protection is largely attributed to the poor durability of humoral immune responses, driven in part by impaired memory B cell formation, suboptimal antibody affinity maturation, and immune evasion by the virus [4,48]. Consequently, reinfection is common across all age groups, particularly in infants and older adults. These challenges underscore the need for innovative strategies that promote durable immune imprinting, especially in early life. Approaches such as maternal vaccination, infant immunoprophylaxis (e.g., with long-acting monoclonal antibodies), and booster vaccine schedules must be assessed not only for their ability to confer acute protection during high-risk periods but also for their impact on long-term immune memory and modulation of RSV disease trajectory [53,152,153]. A better understanding of how different immunization strategies shape lifelong immunity will be critical for informing public health policy and vaccine deployment.

### 7.4. Emerging Technologies

Recent advances in vaccinology have introduced innovative approaches for the prevention and treatment of RSV infection. Among these, mRNA vaccines—particularly those encoding the stabilized prefusion F protein—have garnered attention due to their rapid adaptability, strong immunogenicity, and encouraging safety and efficacy profiles in both animal models and early-phase human trials [154]. In parallel, nanoparticle-based delivery systems have been developed to enable the co-delivery of antigens and adjuvants, thereby improving mucosal targeting and enhancing cellular immune responses [155,156]. Moreover, the integration of system vaccinology and single-cell transcriptomics has opened new avenues for dissecting the immune response at high resolution, allowing the identification of molecular signatures predictive of protective or pathogenic outcomes [157]. These tools hold promise for improving patient stratification and guiding the design of personalized immunotherapeutic strategies.

## 8. Contribution to the Existing Literature

While previous reviews such as that by Hu et al. [158] have comprehensively addressed the virological and cellular mechanisms underlying RSV infection, including viral replication, protein function, and host–pathogen interactions at the molecular level, the present review provides a distinct and complementary perspective by focusing on the immunopathogenesis of RSV and its clinical implications. Specifically, we underscore how severe disease often stems not from uncontrolled viral replication alone, but from dysregulated host immune responses, including Th2/Th17 polarization, impaired interferon signaling, immune checkpoint activation, and inflammasome overactivation. In doing so, we integrate emerging evidence on age-specific immunological vulnerabilities—such as neonatal immune immaturity and immunosenescence in the elderly—that shape disease severity and response to infection. Moreover, this review highlights immunomodulation as a promising and underexplored therapeutic frontier, presenting novel targets such as IL-6, IL-1β, the JAK-STAT pathway, and PD-1/PD-L1 as potential interventions. By bridging detailed immunological mechanisms with recent clinical advances in monoclonal antibodies and maternal vaccination, our work adds a translational dimension to RSV research and offers a conceptual framework for future host-directed therapies.

## 9. Conclusions

RSV represents a persistent global health challenge, not only because of its high transmissibility and recurrent infections, but also due to its ability to manipulate and dysregulate host immune responses. This review underscores that RSV pathogenesis is driven as much by viral factors as by the host’s maladaptive immune activation, particularly excessive cytokine release, Th2/Th17 polarization, and impaired IFN signaling. The virus’s immune evasion strategies—ranging from interferon suppression to dendritic cell dysfunction and antibody decoy mechanisms—contribute to viral persistence, immunopathology, and long-term respiratory sequelae. Age-specific vulnerabilities in infants and older adults further complicate disease outcomes and therapeutic approaches. Recent breakthroughs in monoclonal antibodies, vaccine development, and emerging immunomodulatory therapies targeting cytokine pathways, inflammasomes, and innate immune receptors offer new hope. However, future efforts must aim to refine these interventions to strike a delicate balance between effective viral clearance and mitigation of immune-mediated tissue damage. Tailoring strategies to age and risk profiles, while enhancing our understanding of RSV-host immune interactions, will be pivotal in reducing the burden of this complex and evolving pathogen.

## Data Availability

No new data were created or analyzed in this study. Data sharing is not applicable to this article.
